# PD-1 inhibition plus platinum-based chemotherapy (PBC) or PBC alone in the first-line treatment of locally advanced or metastatic pulmonary lymphoepithelioma-like carcinoma

**DOI:** 10.3389/fimmu.2022.1015444

**Published:** 2022-09-29

**Authors:** Xuanye Zhang, Yixin Zhou, Hualin Chen, Chen Chen, Zuan Lin, Li-na He, Wei Du, Tao Chen, Shaodong Hong, Sha Fu

**Affiliations:** ^1^ State Key Laboratory of Oncology in South China, Guangzhou, China; ^2^ Collaborative Innovation Center for Cancer Medicine, Guangzhou, China; ^3^ Department of Medical Oncology, Sun Yat-sen University Cancer Center, Guangzhou, China; ^4^ Department of Very Important Person (VIP) Region, Sun Yat-sen University Cancer Center, Guangzhou, China; ^5^ Department of Pulmonary Oncology, Affiliated Hospital of Guangdong Medical University, Zhanjiang, China; ^6^ Department of Radiation Oncology, Sun Yat-sen University Cancer Center, Guangzhou, China; ^7^ Department of Clinical Research, Sun Yat-sen University Cancer Center, Guangzhou, China; ^8^ Department of Nuclear Medicine, Sun Yat-sen University Cancer Center, Guangzhou, China; ^9^ Department of Cellular and Molecular Diagnostics Center, Sun Yat-Sen Memorial Hospital, Sun Yat-Sen University, Guangzhou, China; ^10^ Guangdong Provincial Key Laboratory of Malignant Tumor Epigenetics and Gene Regulation, Sun Yat-sen Memorial Hospital, Sun Yat-sen University, Guangzhou, China

**Keywords:** pulmonary lymphoepithelioma-like carcinoma, PD-1, PD-L1, immunotherapy, chemotherapy

## Abstract

**Background:**

Pulmonary lymphoepithelioma-like carcinoma (PLELC) is a distinctive subtype of non-small cell lung carcinoma that was not well presented in clinical studies. The management of advanced PLELC remains an important, unmet need due to the paucity of high-grade evidence. Herein, we carried out a multicenter, retrospective study to assess the effectiveness and tolerability of PD-1/PD-L1 inhibitor plus chemotherapy versus chemotherapy alone for patients with advanced PLELC in the first-line setting.

**Patients and Methods:**

This retrospective study enrolled patients with advanced PLELC receiving first-line treatment with PD-1 inhibition plus chemotherapy (IO-Chemo group) or chemotherapy alone (Chemo group) in three medical centers in China. The survival outcomes, efficacy, and safety profile were investigated. The primary endpoint was progression-free survival (PFS). Secondary endpoints included objective response rate (ORR), overall survival (OS), and adverse events (AEs).

**Results:**

A total of 133 patients were enrolled. PFS was significantly longer in the IO-Chemo group (median 12.8 months [95% CI 5.2-20.4]) than that in the Chemo group (median 7.7 months [95% CI 6.8-8.6]; hazard ratio [HR] 0.48 [95% CI 0.31-0.74]; P=0.001). ORR was 74.5% (95% CI, 63.0-86.1) in the IO-Chemo group and 34.6% (95% CI, 24.1-45.2) in the Chemo group (P<0.001). The median OS was not reached in the IO-Chemo group versus 35.7 months (95% CI 26.7-44.8) in the Chemo group (HR 0.47 [95% CI 0.20-1.07]; P=0.065). Multivariate analysis revealed that PD-1/PD-L1 inhibitor combination was independently associated with longer PFS (HR 0.40 [95% CI 0.25-0.63]; P<0.001). Grade 3 or higher AEs occurred in 36 (65.5%) patients in the IO-Chemo group and 56 (71.8%) patients in the Chemo group, respectively.

**Conclusions:**

In patients with advanced PLELC, adding PD-1/PD-L1 inhibitor to platinum-based chemotherapy significantly increased PFS and ORR with a tolerable safety profile.

## Introduction

Pulmonary lymphoepithelioma-like carcinoma (PLELC) is rare and distinctive type of non-small-cell lung carcinoma (NSCLC), representing 0.9% of all lung malignancies (1). It is etiologically attributed to Epstein-Barr virus (EBV) and has similar histological properties with undifferentiated nasopharyngeal carcinoma (NPC) (2, 3). Previously categorized as “other and unclassified carcinomas”, PLELC was reclassified into “squamous cell carcinoma” in the 2021 World Health Organization (WHO) classification of thoracic tumors (4). However, no consensus has been reached on the appropriate histologic classification of PLELC.

Most of the PLELC patients are diagnosed at early stage and radical resection is the preferred treatment for these patients (5–8). The management of stage IIIA-IIIC PLELC is clinically challenging and need to be discussed in a multidisciplinary setting (9–11). However, a subset of PLELC patients may present with advanced disease who are not amenable for local therapies, resulting in significant morbidity or mortality. For first-line palliative chemotherapy, the most prevalent regimens are platinum plus an anti-metabolite agent such as 5-fluorouracil, pemetrexed, taxanes, or gemcitabine (10, 12). However, due to the rarity of PLELC, there are no established management guidelines for locally advanced or metastatic PLELC, thus remaining an important, unmet need. Our and other researchers’ findings suggested that platinum plus taxanes (TP) or gemcitabine (GP) chemotherapy provide more clinical benefit than pemetrexed plus platinum (AP) in the first-line treatment of advanced PLELC (12–14).

At molecular level, we have previously unveiled that PLELC lacks druggable driver mutations. The altered signaling pathways, patterns of somatic mutations and copy number variations of PLELC resembles those of NPC, but not other lung cancer subtypes, i.e. squamous cell lung carcinoma, lung adenocarcinoma, and small-cell lung carcinoma. As such, targeted therapy options for PLELC patients are scarce.

Programmed cell death 1 (PD-1)/programmed cell death-ligand 1 (PD-L1) blockade therapy has demonstrated efficacy in NSCLC. Specifically, PD-1/PD-L1 inhibitors plus platinum-based chemotherapy prolongs patients’ survival irrespective of PD-L1 level in both non-squamous and squamous NSCLC (15–20). However, clinical trials of anti-PD-1/PD-L1 therapy in NSCLC rarely enrolled patients with PLELC because of its rarity and the controversy of its classification. Evidence showed that the proportion of PD-L1-positive tumor cells in PLELC is higher than that in lung adenocarcinoma or squamous cell lung carcinoma (21–23), offering potential for anti-PD-1/PD-L1 therapy for this distinctive subtype of NSCLC. To date, the utilization of PD-1/PD-L1 inhibitors for patients with advanced PLELC was described in a few case reports or small case series.

Considering the under representation of PLELC in clinical trials, the controversy of optimal first-line palliative therapy and the potential effectiveness of anti-PD-1/PD-L1 immunotherapy in PLELC, we conducted a multi-institutional, retrospective study to compare PD-1 inhibition plus GP/TP chemotherapy with GP/TP chemotherapy alone in patients with advanced PLELC.

## Patients and methods

Between January, 2010 and December, 2021, patients with advanced PLELC who received anti-PD-1/PD-L1 therapy plus GP/TP regimens or GP/TP chemotherapy alone in the first-lien setting were screened from three academic medical centers in China (Sun Yat-sen University Cancer Center, Sun Yat-Sen Memorial Hospital and Affiliated Hospital of Guangdong Medical University). Eligible patients should meet the following criteria: (1) histologically diagnosed as PLELC; (2) excluding lung metastasis from nasopharyngeal carcinoma; (3) stage IIIB or IIIC who were not amenable to radical radiotherapy/surgery or stage IV according to the 8th edition of the Lung Cancer stage classification system, and (4) received at least one cycle of the above-mentioned therapies. The pathology diagnoses were made in each respective institution and double confirmed by a third pathologist (Dr. Sha Fu, Sun Yat-sen Memorial Hospital, Sun Yat-sen University). Clinical information was collected from patient medical records. Patient demographic, tumor and *in situ* treatment information were recorded.

Response to treatment was assessed by the physicians at each participating center, according to the Response Evaluation Criteria in Solid Tumors (RECIST), version 1.1. Response was categorized as: complete response (CR), partial response (PR), progressive disease (PD), and stable disease (SD). The objective response rate (ORR) was defined as the proportion of patients with CR or PR and the disease control rate (DCR) was defined as the proportion that achieved CR, PR or SD. Progression-free survival (PFS) was defined as the time from the initiation of treatment to PD or death. Overall survival (OS) was measured from the initiation of treatment until the date of death. Adverse events (AEs) were retrospectively evaluated, per the National Cancer Institute Common Terminology Criteria for Adverse Events (CTCAE), version 5.0.

This study was approved by the Sun Yat-sen University Cancer Center Institutional Review Board (No. B2020-402-01). Informed consent was not required due to the retrospective nature. This study adhered to the principles of the Declaration of Helsinki.

### Statistical analysis

The primary objective of the study was to compare the PFS between PD-1/PD-L1 inhibitor plus chemotherapy group and chemotherapy alone group. Secondary end points included OS, ORR, DCR, as well as the incidence and severity of AEs. Baseline characteristics and treatment-related AEs were tabulated and summarized. Pearson’s χ^2^-test or Fisher’s exact test was used to compare between-group differences for categorical variables. The Kaplan-Meier methodology was used for survival analysis. The survival rate between the two groups were analyzed using the log-rank test. Investigation of the effect of PD-1/PD-L1 inhibitor and other baseline characteristics on treatment outcomes was performed using univariate and multivariate Cox regression analysis. A 2-tailed P value of ≤0.05 was deemed significant. All statistical analyses were performed using SPSS software version 22.0 (SPSS Inc., Chicago, IL).

## Results

### Patients

A total of 133 patients were eligible. Baseline characteristics are summarized in **Table 1**. For the entire cohort, the median age was 52 (range, 29-73) years. There were more females than males (57.9% vs. 42.1%, respectively). All the included patients had an ECOG performance status of 0 or 1. One hundred (75.2%) patients had no history of smoking. According to the TNM staging system, 116 (87.2%) patients were diagnosed at stage IV disease and 17 (12.8%) at IIIB-IIIC. The most common anatomic sites of metastasis included bone (38.3%) and pleura (30.8%). Seventy-one (53.4%) patients had their tumors tested for classical EGFR mutation and ALK translocation status and none were positive. All the cases were positive for EBERs. Baseline EBV DNA was available in 64 (48.2%) patients and the median titer was 1.09×10^4^ copies/mL (range, 0 to 3.46×10^6^ copies/mL). Fifty-five (41.4%) patients received PD-1/PD-L1 inhibitor plus GP or TP regimens (IO-Chemo group) and 78 (58.6%) received GP/TP chemotherapy alone (Chemo group). Between-group baseline characteristics were similar. Twelve patients in the IO-Chemo group had results of PD-L1 expression (22C3). All of them were PD-L1-positive (PD-L1 TPS of ≥1%) and 8 (66.7%) had a PD-L1 TPS of ≥50%.

**Table 1:** Baseline characteristics of the included patients.

	Total, n (% ) (n = 133)	PD-1/PD-L1 inhibitor^a^+GP/TP, n (%) (n = 55)	GP/TP, n (%) (n = 78)	P value^b^
Median age (range), years	52 (29-73)	50 (32-73)	52 (29-70)	
Sex				0.955
Male	56 (42.1)	23 (41.8)	33 (42.3)	
Female	77 (57.9)	32 (58.2)	45 (57.7)	
Stage				0.299
IIIB-IIIC	17 (12.8)	9 (16.4)	8 (10.3)	
IV	116 (87.2)	46 (83.6)	70 (89.7)	
ECOG performance status				0.717
0	87 (65.4)	35 (63.6)	52 (66.7)	
1	46 (34.6)	20 (36.4)	26 (33.3)	
Smoking status				0.502
Current or former	33 (24.8)	12 (21.8)	21 (26.9)	
Never	100 (75.2)	43 (78.2)	57 (73.1)	
Metastasis site				
Lung	41 (30.8)	16 (29.1)	25 (32.1)	0.716
Live	37 (27.8)	13 (23.6)	24 (30.8)	0.366
Bone	51 (38.3)	22 (40.0)	29 (37.2)	0.742
Pleura	41 (30.8)	22 (40.0)	19 (24.4)	0.054
Distant lymph nodes	19 (14.3)	9 (16.4)	10 (12.8)	0.565
Others	18 (13.5)	7 (12.7)	11 (14.1)	0.819
EGFR or ALK genomic aberrations				0.199
Yes	0 (0)	0 (0)	0 (0)	
No	71 (53.4)	33 (60.0)	38 (48.7)	
Unknown	62 (46.6)	22 (40.0)	40 (51.3)	
Baseline EBV DNA level				0.098
<1×10^4^ copies/mL	32 (24.1)	15 (27.3)	17 (21.8)	
≥1×10^4^ copies/mL	32 (24.1)	8 (14.5)	24 (30.8)	
Unknown	69 (51.9)	32 (58.2)	37 (47.4)	
Chemotherapy regimen				
GP	24 (18.0)	7 (12.7)	17 (21.8)	0.181
TP	109 (82.0)	48 (87.3)	61 (78.2)	

a Including pembrolizumab (n = 19), sintilimab (n = 11), tislelizumab (n = 8), toripalimab (n = 7), camrelizumab (n = 7), nivolumab (n = 1) and durvalumab (n = 2).

b Determined using the χ2 test.

GP, gemcitabine plus platinum; TP, taxanes plus platinum; PD-1, programmed cell death protein-1; PD-L1, programmed cell death-ligand 1; ECOG, Eastern Cooperative Oncology Group; EGFR, epidermal growth factor receptor; ALK, anaplastic lymphoma kinase; EBV, Epstein-Barr virus.

### Effectiveness

At data collection date of August, 2022, the median follow-up for the entire cohort was 23.1 (range, 2.9-73.0) months (19.7 [range, 5.6-38.4] months for the IO-Chemo group and 29.3 [range, 2.9-73.0] months for the Chemo group). The median (range) cycle of chemotherapy was 4 (1-6) in both groups. Following induction treatment cycles, 45 (81.8%) patients in the IO-Chemo group received maintenance therapy with PD-1/PD-L1 inhibitor at the discretion of the treating physician and patients in the Chemo group underwent best supportive care.

**Figure 1:**
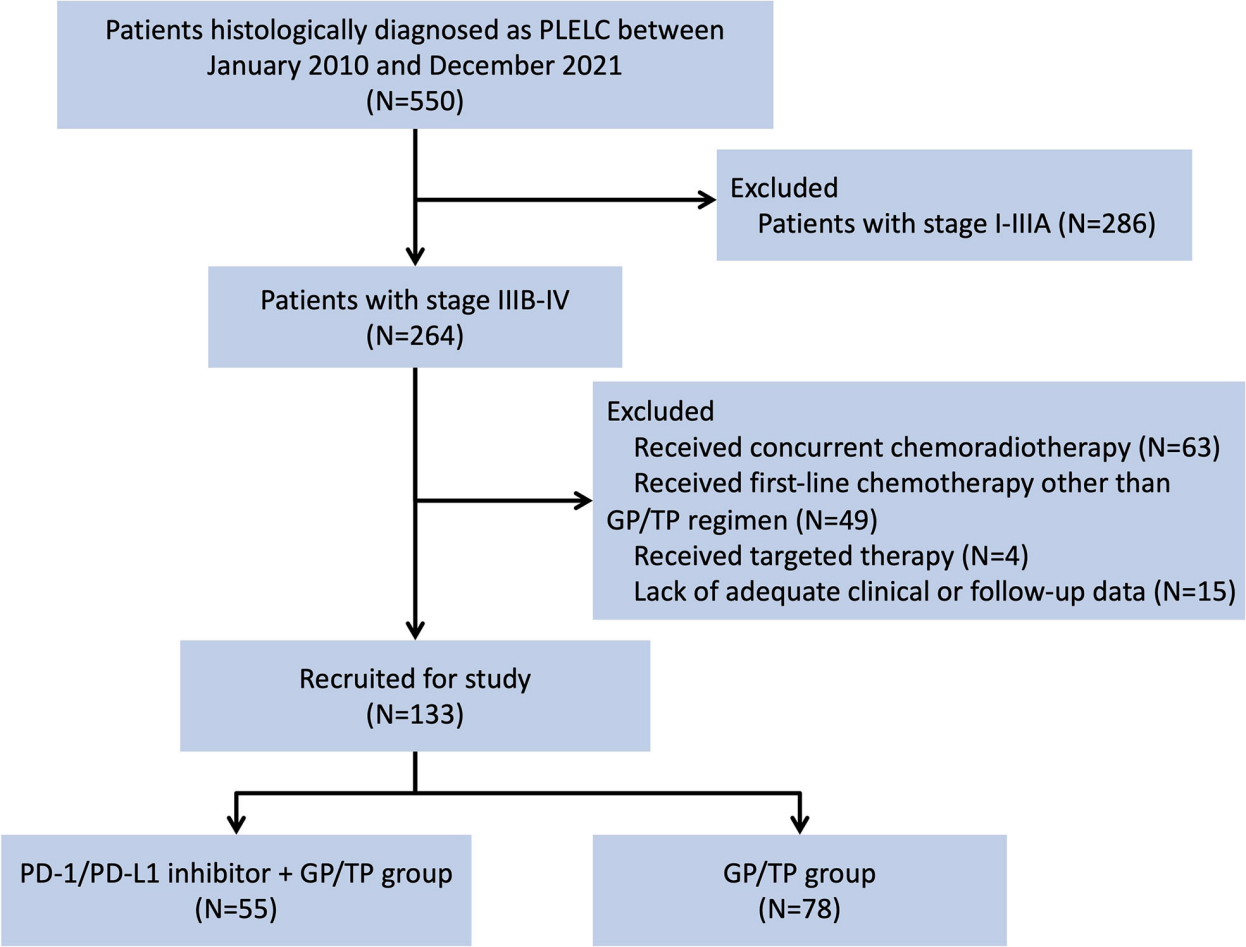
Flow chart depicting patient deposition. PLELC, pulmonary lymphoepithelioma-like carcinoma; GP, gemcitabine plus platinum; TP, taxanes plus platinum; PD-1, programmed cell death protein-1; PD-L1, programmed cell death-ligand 1.

By the cut-off date, 29 (52.7%) patients in the IO-Chemo group and 63 (80.1%) patients in the Chemo group had events of progression or death. The median PFS was 12.8 months (95% CI 5.2-20.4 months) in the IO-Chemo group and 7.7 months (95% CI 6.8-8.6 months) in the Chemo group (HR 0.48 [95% CI 0.31-0.74]; P=0.001) (**Table 2**; **Figure 1**). The estimated proportion of patients who were progression-free and stayed alive at 12 months was 54.5% in the IO-Chemo group and 23.8% in the Chemo group.

**Table 2:** Univariate analysis of PFS and OS.

Variables	PFS		OS	
	HR (95% CI)	P-value	HR (95% CI)	P-value
Age, years		0.325		0.210
<50	Reference		Reference	
≥50	1.23 (0.81-1.89)		1.54 (0.78-3.04)	
Sex		0.878		0.820
Female	Reference		Reference	
Male	1.03 (0.68-1.57)		1.07 (0.57-2.03)	
Stage		0.100		0.401
IIIB-IIIC	Reference		Reference	
IV	1.89 (0.87-4.10)		1.65 (0.51-5.38)	
ECOG performance status		0.257		0.013
0	Reference		Reference	
1	1.28 (0.83-1.97)		2.21 (1.17-4.18)	
Smoking status		0.543		0.658
Never	Reference		Reference	
Current or former	1.16 (0.72-1.88)		1.18 (0.57-2.42)	
Liver metastasis		0.003		<0.001
No	Reference		Reference	
Yes	1.95 (1.24-3.07)		4.13 (2.08-8.21)	
Baseline EBV DNA level		<0.001		0.002
<1×10^4^ copies/mL	Reference		Reference	
≥1×10^4^ copies/mL	2.37 (1.26-4.45)		6.54 (2.02-21.09)	
Unknown	3.06 (1.75-5.37)		4.99 (1.68-14.83)	
Chemotherapy regimen		0.262		0.082
GP	Reference		Reference	
TP	1.37 (0.79-2.39)		0.54 (0.27-1.01)	
PD-1/PD-L1 inhibitor combination		0.001		0.065
No	Reference		Reference	
Yes	0.48 (0.31-0.74)		0.47 (0.20-1.07)	

PFS, progression-free survival; OS, overall survival; HR, hazard ratio; ECOG, Eastern Cooperative Oncology Group; EBV, Epstein-Barr virus; GP, gemcitabine plus platinum; TP, taxanes plus platinum; PD-1, programmed cell death protein-1; PD-L1, programmed cell death-ligand 1.

**Figure 2:**
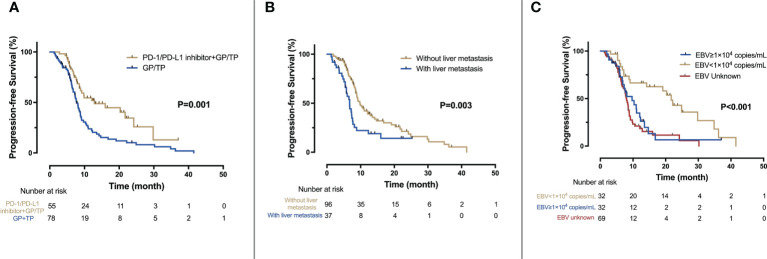
Kaplan-Meier analysis of PFS. **(A)** PFS for the IO-Chemo group and the Chemo group; **(B)** PFS for patients with and without liver metastasis; **(C)** PFS according to the level of baseline EBV DNA.

The median OS for the overall population was 38.6 months (95% CI 32.9-44.4 months) (**Figure 2A**). A total of 38 patients ceased, with 5 in the IO-Chemo group and 33 in the Chemo group. The median OS was not reached in the IO-Chemo group versus 35.7 months (95% CI 26.7-44.8 months) in the Chemo group (HR 0.47 [95% CI 0.20-1.07]; P=0.065) (**Table 2**; **Figure 2B**).

Efficacy data are presented in **Table 3**. Forty-one (74.5%) patients in the IO-Chemo group and 27 (34.6%) in the Chemo group achieved PR while 14 (25.5%) in the IO-Chemo group and 45 (57.7%) in the Chemo group achieved SD as their best response. No patients achieved CR in both groups. The ORR was 74.5% (95% CI, 63.0–86.1) in the IO-Chemo group and 34.6% (95% CI, 24.1-45.2) (P<0.001) in the Chemo group. The DCR was similar between the IO-Chemo group and Chemo group (100% vs 92.3%, P=0.093). The median duration of response (DoR) was 16.0 months (range, 4.8+ to 37.0+) in the IO-Chemo group and 8.8 months (range, 4.6+ to 41.5) in the Chemo group. For patients who achieved SD, the median duration of SD was 8.5 months (range 2.8 to 21.7) in the IO-Chemo group and 7.6 months (range, 1.9 to 24.9) in the Chemo group. (Plus signs in the ranges denotes the absence of a progressive disease at the time of the latest disease evaluation).

**Table 3:** Tumor response with PD-1/PD-L1 inhibitor plus GP/TP versus GP/TP.

Characteristics	PD-1/PD-L1 inhibitor+GP/TP (n = 55)	GP/TP (n = 78)	Total (n = 133)
Best overall response, n (%)			
PR	41 (74.5)	27 (34.6)	68 (51.1)
SD	14 (25.5)	45 (57.7)	59 (44.4)
PD	0 (0)	6 (7.7)	6 (4.5)
ORR			
ORR, % (95% CI)	74.5 (63.0-86.1)	34.6 (24.1-45.2)	51.1 (42.6-59.6)
P value^a^	<0.001		
DCR			
DCR, % (95% CI)	100	92.3 (86.4-98.2)	95.6 (92.0-99.0)
P value^b^	0.093		

^a,b^Determined using the χ2 test.

GP, gemcitabine plus platinum; TP, taxanes plus platinum; PD-1, programmed cell death protein-1; PD-L1, programmed cell death-ligand 1; PR, partial response; SD, stable disease; PD, progressive disease; ORR, objective response rate; DCR, disease control rate, CI, confidence interval.

### Adverse events

Adverse events of any cause and irrespective of attribution to treatment was noted in 54 (98.2%) patients in the IO-Chemo group versus 76 (97.4%) patients in the Chemo group (**Table 4**). These AEs were of grade 3 or more severity in 36 (65.5%) patients in the IO-Chemo group and 56 (71.8%) patients in the Chemo group. In both groups, the most common grade 3 or more severity AEs were hematologic, including neutropenia (25 [45.5%] in the IO-Chemo group vs 39 [50.0%] in the Chemo group), anemia (17 [30.9%] vs 20 [25.6%]) and thrombocytopenia (4 [7.3%] vs 7 [9%]). Two patients experienced treatment-related AEs leading to death in the Chemo group and no treatment-related deaths were documented in the IO-Chemo group.

**Table 4:** Treatment-related adverse events in both groups.

Event, n (%)	PD-1/PD-L1 inhibitor+GP/TP (n = 55)		GP/TP (n = 78)	
	Any grade	Grade≥3	Any grade	Grade≥3
Any^a^	54 (98.2)	36 (65.5)	76 (97.4)	56 (71.8)
Neutropenia	43 (78.2)	25 (45.5)	62 (83.3)	39 (50.0)
Anaemia	43 (78.2)	17 (30.9)	60 (76.9)	20 (25.6)
Thrombocytopenia	22 (40.0)	4 (7.3)	32 (41.0)	7 (9.0)
Fatigue	42 (76.4)	0	65 (83.3)	2 (2.6)
Nausea	31 (56.4)	0	48 (61.5)	0
Peripheral neuropathy	23 (41.8)	1 (1.8)	29 (37.2)	2 (2.6)
Rash	18 (32.7)	1 (1.8)	17 (21.8)	0
Vomiting	9 (16.4)	0	16 (20.5)	0
ALT increased	8 (14.5)	1 (1.8)	14 (17.9)	1 (1.3)
AST increased	7 (12.7)	1 (1.8)	14 (17.9)	1 (1.3)
Blood creatinine increased	7 (12.7)	0	16 (20.5)	0

The table shows adverse events occurring in at least 10% of patients in any group.

a Worst per patient.

PD-1, programmed cell death protein-1; PD-L1, programmed cell death-ligand 1; GP, gemcitabine plus platinum; TP, taxanes plus platinum; ALT, alanine aminotransferase; AST, aspartate aminotransferase.

According to investigator assessment, 14 of 51 (25.5%) patients in the IO-Chemo group had immune-related AEs (irAEs) (**Table 5**). Hypothyroidism, rash and stomatitis were the most common irAEs. Most of the irAEs were grade 1-2 and only 2 (3.9%) patients developed grade 3 irAEs (one with grade 3 rash and one with grade 3 hepatitis). The patient with grade 3 hepatitis and another patient with grade 2 pneumonitis experienced permanent discontinuation of PD-1/PD-L1 blockade treatment.

**Table 5:** Immune-related adverse events in the IO-Chemo group.

Event	No. (%)		
	Grade 1-2	Grade 3	Total
Any^a^	12 (21.8)	2 (3.6)	14 (25.5)
Hypothyroidism	9 (16.4)	0	9 (16.4)
Rash	8 (14.5)	1 (1.8)	9 (16.4)
Stomatitis	4 (7.3)	0	4 (7.3)
Hypocortisolism	3 (5.5)	0	3 (5.5)
Reactive capillary haemangiomas	3 (5.5)	0	3 (5.5)
Hepatitis	2 (3.6)	1 (1.8)	3 (5.5)
Infusion reaction	2 (3.6)	0	2 (3.6)
Pneumonitis	2 (3.6)	0	2 (3.6)
Hyperthyroidism	1 (1.8)	0	1 (1.8)

a Worst per patient.

### Univariate and multivariate Cox regression analyses of PFS and OS

**Figure 3:**
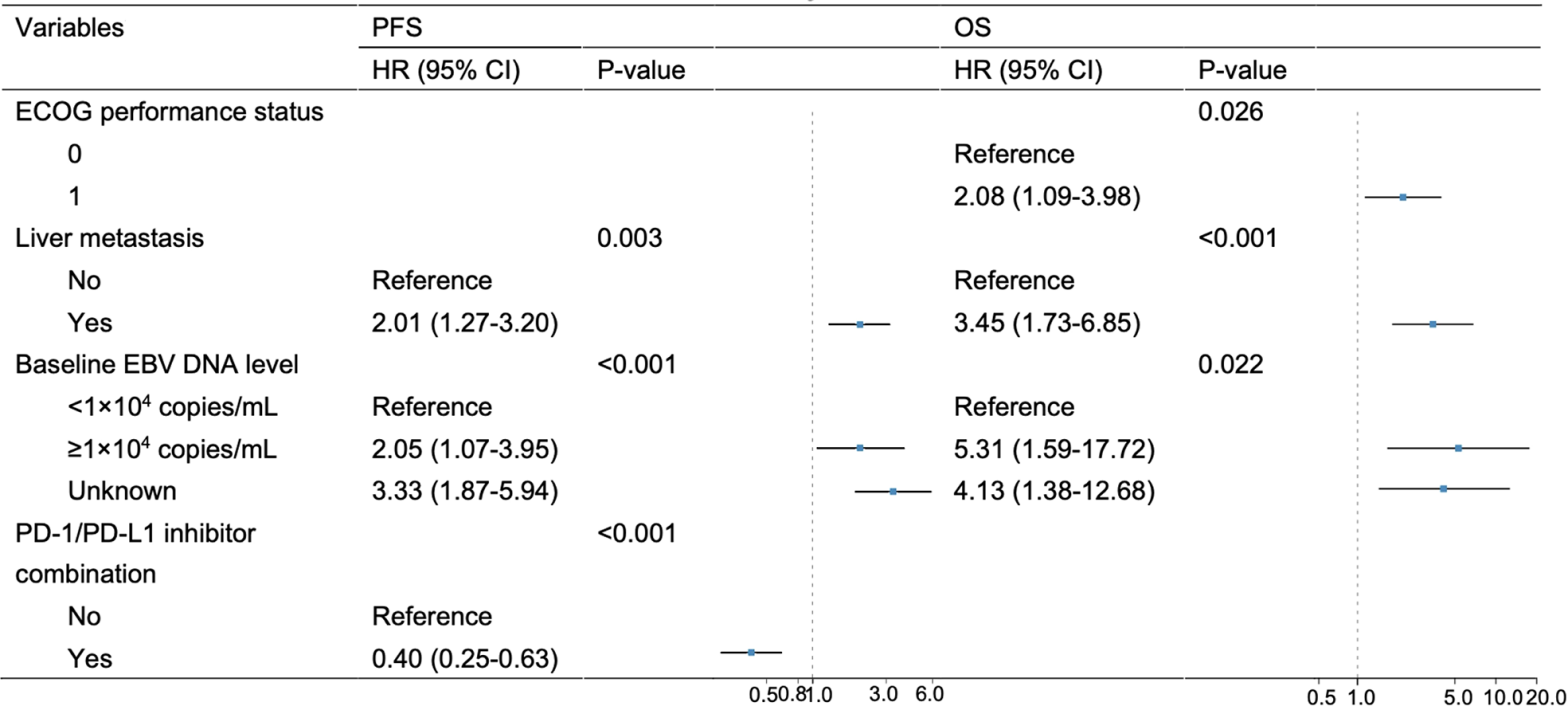
Multivariate analysis of PFS and OS. Variables achieving P < 0.05 in univariate analysis were entered into multivariate analysis.

Univariate and multivariate analyses are presented in **Table 2** and **Figure 3**. Univariate analysis revealed that patients without liver metastasis (P=0.003), having low baseline EBV DNA load (<1×10^4^ copies/mL) (P<0.001) and receiving PD-1/PD-L1 inhibitor-containing treatment (P=0.001) had significantly longer PFS (**Figure 2**). Variables that achieved statistical significance in univariate analysis were entered into multivariate analysis. Multivariate analysis identified liver metastasis (HR=2.01; P=0.003), baseline EBV DNA level (HR=2.05; P<0.001) and PD-1/PD-L1 inhibitor combination (HR=0.40; P<0.001) were independently associated with PFS.

**Figure 4:**
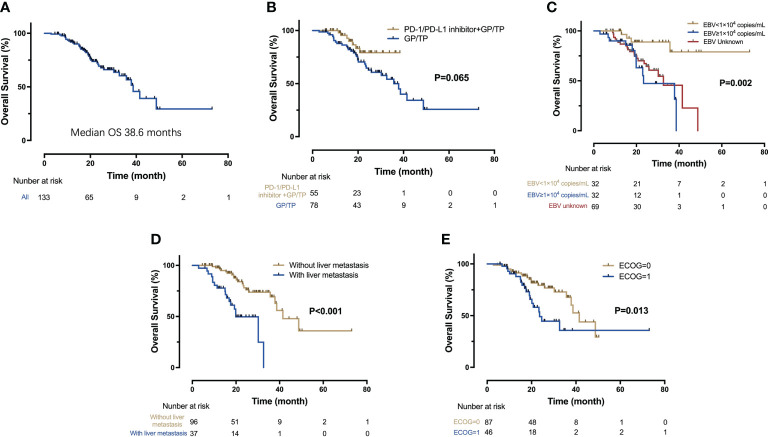
Kaplan-Meier analysis of OS. **(A)** OS for the entire cohort (n = 133); **(B)** OS for the IO-Chemo group and the Chemo group; **(C)** OS according to the level of baseline EBV DNA; **(D)** OS for patients with and without liver metastasis; **(E)** OS according to the ECOG performance status.

ECOG performance status of 0 (P=0.013), no liver metastasis (P<0.001) and baseline EBV DNA level of lower than 10,000 copies/mL (P=0.002) were associated with longer OS per univariate analysis (**Figure 4**). Multivariate analysis confirmed that ECOG performance status (HR=2.08; P=0.026), liver metastasis (HR=3.45; P<0.001) and baseline EBV DNA load (HR=5.31, P=0.022) were independent prognostic factors for OS.

## Discussion

Herein, we present our real-world study of PD-1 pathway inhibition plus platinum-based chemotherapy for patients with PLELC in the first line setting. To our knowledge, it is the largest such study up to now, and it is the first muti-institutional study to compare PD-1/PD-L1 inhibitor plus chemotherapy with chemotherapy alone for PLELC. Given the rarity of PLELC and the lack of prospective data, this study help impart clinical insight into the management of advanced PLELC with PD-1/PD-L1 pathway inhibition.

The findings of this study mirrored those from the randomized studies of locally advanced lung adenocarinoma and squamous cell lung carcinoma. In our patient population, adding an anti-PD-1 therapy to chemotherapy resulted in a 52% reduction in the risk of disease progression or death compared with chemotherapy alone in patients with untreated, advanced PLELC. The benefit of introducing immunotherapy also was observed in tumor response, as evident by improved ORR and DOR.

Although classified as “squamous cell carcinoma” in the 2021 WHO classification of lung tumors (4), the pathologic classification of PLELC remains highly controversial. The epidemiological characteristics, histopathologic features, genetic landscape and clinical outcomes of PLELC are obviously different from squamous carcinoma lung cancer or other subtypes of NSCLC (2, 8, 13, 24–27). PLELC should be considered as a distinct entity in lung malignancy. Typical driver mutations reported in lung adenocarcinoma are rarely detected in PLELC (13, 28–30), indicating that the molecular mechanism of its tumorigenesis is different from other types of NSCLC and targeted therapy is not a suitable treatment option for PLELC. Similarly, of the 67 tested tumors in our study, all were negative for EGFR mutation or ALK rearrangement.

Thus far, no consensus has been made on the front-line management of advanced PLELC and the decided chemotherapy regimen is empirical. Like NPC, TP or GP chemotherapy yields higher response rate than AP regimen does (12–14). Despite the high response rate with chemotherapy in PLELC, the reported median PFS for front-line GP or TP regimen is only about 7-10 months, hindering patients’ survival improvement (12–14). With the rise of immunotherapies, PD-1/PD-L1 inhibitor plus chemotherapy has become the preferred front-line treatment for patients with advanced NSCLC (15–20). However, due to the rarity and the ambiguous histological classification, PLELC patients were barely enrolled in these immunotherapy-related clinical trials. Besides the close link with EBV infection, tumor infiltrating lymphocytes and PD-L1 over-expression were often observed in PLELC (21–23, 31). As previously reported, 63.3–75.8% of the PLELC had PD-L1 positivity, which was higher than that in other subtypes of NSCLC (32) but similar to that in NPC (33). Of the 12 detected patients in our study, more than 60% had PD-L1 TPS of ≥50%. Currently, two phase III studies demonstrated that a PD-1 inhibitor plus GP chemotherapy significantly prolonged PFS for patients with advanced NPC compared with GP chemotherapy alone (33, 34). Collectively, this evidence provides a potential for anti-PD-1/PD-L1 therapy in PLELC. Several case reports also implied that anti-PD-1/PD-L1 monotherapy was active in advanced PLELC in the second- or third-line settings (35–39). Recently, a retrospective study enrolling 19 advanced PLELC patients receiving immunotherapy (as a single agent or plus chemotherapy) demonstrated the encouraging effectiveness of front-line ICI therapy (40). However, the sample sizes of these studies are too small to draw robust conclusion. In the current study, with the largest ICI-treated, advanced PLELC cohort, a significant improvement of PFS was demonstrated in PD-1 inhibitor plus chemotherapy group compared with chemotherapy alone group (median PFS, 12.8 months vs 7.7 months; HR=0.48, P=0.001). The ORR was significantly improved (74.5% vs 34.6%, P<0.001) and DoR was also longer in the IO-Chemo group. Although the OS data was immature in the IO-Chemo group, preliminary data indicated that adding PD-1/PD-L1 inhibitor to chemotherapy tended to prolong OS (HR=0.47, P=0.065). Moreover, multivariate analysis showed that the addition of PD-1/PD-L1 was an independent protective factor for PFS, after adjusting for known prognostic factors. Taken together, our results reveal the benefits of anti-PD-1/PD-L1 immunotherapy for patients with PLELC and support further assessment of this combination approach in prospective studies.

PD-1/PD-L1 blockade therapy plus chemotherapy was generally well tolerated during this study. AEs noted herein was consistent with the safety profiles of GP/TP regimen ± anti-PD-1/PD-L1 antibodies reported in other clinical trials (18, 33, 34, 41). In both groups, most treatment-related AEs were hematologic ones, which were mainly attributed to chemotherapy regimens. As expected, the incidence of severe irAEs in patients with PD-1/PD-L1 inhibitor-containing treatment was low and the irAEs were manageable in general. Notably, a frequent adverse event, peripheral neuropathy, considered to be related to taxanes, was observed in more than 1/3 of patients in this study. Though mostly classified to grade 1-2, the peripheral neuropathy deserves vigilance in clinical practice.

Previous studies have revealed a potential association between baseline EBV DNA load and survival of PLELC patients with surgery, radiotherapy or chemotherapy (12, 42). So far, no studies have investigated the prognostic impact of EBV load in PLELC treated with immunotherapy. In the current study involving patients undergoing ICI-containing therapy, we confirmed that low baseline EBV DNA level strongly correlates with superior PFS and OS in PLELC. The findings imply that plasma EBV DNA could be an effective prognostic indicator in advanced PLELC patients, which was very similar to that in NPC patients. However, due to the retrospective design of this study, a substantial number of patients lacked baseline EBV DNA data. Considering its predictive value, we recommend incorporating baseline EBV DNA copy number into regular clinical practice in all PLELC patients.

We acknowledged that the retrospective nature, the lack of biomarkers (PD-L1, tumor mutational burden, tumor microenvironment indicators, et al.) within this study, the relatively small sample size were major limitations. However, our studies also have several strengths, including data from multiple centers and real-world setting, the largest number of ICI-treated PLELC patients enrolled. Given the wide application of ICI plus platinum-based chemotherapy for advanced NSCLC, we believe our findings are clinically relevant, informative and innovative.

## Conclusions

The addition of PD-1/PD-L1 inhibitor to platinum-based chemotherapy as first-line treatment for patients with advanced PLELC has improved efficacy compared with chemotherapy alone with a well-tolerated safety profile.

## Data availability statement

The raw data supporting the conclusions of this article will be made available by the authors, without undue reservation.

## Ethics statement

This study was approved by the Sun Yat-sen University Cancer Center Institutional Review Board. Written informed consent was obtained from all participants for their participation in this study.

## Author contributions

Conception and design, SH, and SF;Financial support, SH;Administrative support, CC, ZL, and L-NH; Data analysis and interpretation, XZ, YZ, HC, and CC;All authors contributed to the article and approved the submitted version.

## Funding

This study was funded by grants 8217102281, 81972898, and 81872499 from the National Natural Science Funds of China, 16zxyc04 from the Outstanding Young Talents Program of Sun Yat-sen University Cancer Center, 2019A1515011090 from the Natural Science Foundation of Guangdong Province. The funding sources had no role in the design and conduct of the study, collection, management, analysis, and interpretation of the data, preparation, review, or approval of the manuscript; and decision to submit the manuscript for publication.

## Acknowledgments

We sincerely appreciate all the patients and their family who were included in this retrospective study.

## Conflict of interest

The authors declare that the research was conducted in the absence of any commercial or financial relationships that could be construed as a potential conflict of interest.

## Publisher’s note

All claims expressed in this article are solely those of the authors and do not necessarily represent those of their affiliated organizations, or those of the publisher, the editors and the reviewers. Any product that may be evaluated in this article, or claim that may be made by its manufacturer, is not guaranteed or endorsed by the publisher.

## Abbreviations

PLELC, pulmonary lymphoepithelioma-like carcinoma; NSCLC, non-small-cell lung cancer; EBV, Epstein-Barr virus; NPC,nasopharyngeal carcinoma; WHO, World Health Organization; TP, taxanes plus platinum; GP, gemcitabine plus platinum; AP, pemetrexed plus platinum; PD-1, anti-programmed cell death-1; PD-L1, programmed cell death-ligand 1; ECOG, Eastern Cooperative Oncology Group; EBER, Epstein-Barr encoding region; CR, complete response; PR, partial response; SD, stabledisease; PD, progressive disease; ORR, objective response rate; DCR, disease control rate; PFS, progression-free survival; OS, overall survival; AE, adverse event.
